# Supplementary material for: The Effects of Occupation, Education and Dwelling Place on Attitudes towards Animal Welfare in China
Source: Animals (Basel). 2024 Feb 24;14(5):713. doi: 10.3390/ani14050713 (PMC10930921; doi:10.3390/ani14050713)
Supplement: Supplementary file 1 [file animals-14-00713-s001.zip › animals-2841827-supplementary.pdf]

Supplementary Materials

Box S1: Questionnaire.

Location (circle): Rural / Village / City

Province:

1 Do you identify as Chinese? YES (please continue); NO (if no, please do not continue. Thank you for your time)

2 What is your gender? Male; Female; Other; Prefer not to say.

3 How old are you? 18-24; 25-34; 54-44; 45-54; 55-64; >65.

4 Religion: Chinese folks; Atheist; Buddhism; Muslim; Christians; Daoism; Confucianism; Prefer not to say; Other.

5 What is your highest level of education? Elementary school or below; Technical college; Middle school; High school; University undergraduate; University postgraduate.

6 Are you currently employed? Yes; No.

<u>7 If yes</u>, what field do you work in? Administration; Agriculture; Arts; Construction; Education; Finance; Government; Health; Mining; Military; Retail/Sales; Science; Technology; Other.

8 Where do you currently live? Rural; Village; Urban; Other.

9 Have you heard of the phrase 'animal welfare'? Not sure; Never; A few times; Many times.

10 Do you live in harmony with animals? Not at all Slightly; Moderately; Very much; To a great extent.

11 How important is caring for animals to you as a person? Not at all Slightly; Moderately; Very; Extremely.

12 Where did you learn about caring for animals? (Tick all that apply) Formal study**;** Family and friends**;** Media**;** Business**;** My job**;** Government**;** Animal protection organization**;** Social media**;** Farmer**;** Have not heard**;** Other.

13 Do you think that animal care should be taught in schools? Definitely not; Probably not; Possibly; Probably; Definitely.

14Would you be willing to pay more for products from animals that are better cared for? Yes; No

<u>15 If yes,</u> how much more would you be willing to pay for a product from an animal very well cared for compared with the standard product? 5%; 10%; 20%; 50%; 100%; <100%

16 What do you think is the current standard of animal care in China? Very poor; Poor; Satisfactory
Good; Very good.

17 How do you think the standard of animal care in China compares to other countries? Much worse; Somewhat worse; About the same; Better; Much Better.

18 Who do you think is most responsible for the adequate care of animals? (Tick one only) Government; Animal Protection Organizations; Farmers; All of society; People who like animals; People who own animals; Companies that use animals; Other.

19 How important is it that the following animals are cared for?

(Not at all important; Slightly important; Neither important nor unimportant; Somewhat important; Very important.)

19.1 d

20 Why do people take care of farm animals? Indicate how strongly you agree or disagree with the following reasons

(Strongly disagree; Disagree; Neither agree nor disagree; Agree; Strongly agree.)

20.1 It is important for food safety

20.2 It is important for sake of the environment

20.3 It makes me feel good
20.4 My religion tells me to
20.5 It is good for human health
20.6 For sake of the animals
20.7 To improve profit from animals
20.8 To improve product quality or taste
20.9 To be a kind person

21 How important are the following conditions in animal care?

(Not at all important; Slightly important; Neither important nor unimportant; Somewhat important; Very important.)

21.1 Species-relevant nutrition
21.2 Access to drinking water
21.3 A comfortable environment
21.4 Space
21.5 Physical fitness
21.6 Absence of disease or injury
21.7 Control over their environment
21.8 Opportunity to perform natural behaviours
21.9 Absence of fear or distress
21.10 Absence of pain

22 Indicate your level of agreement with the following statements

(Strongly disagree; Disagree; Neither agree nor disagree; Agree; Strongly agree.)

22.1 Farms with animals should be certified by animal protection organizations
22.2 Procedures performed on animals such as ear tags, castrations and tail breaks are acceptable for management
22.3 Transportation time of live animals should be minimized
22.4 Animals on farms should be provided with enjoyable experiences
22.5 It is OK to buy products of animals that have suffered if the product quality is good enough
22.6 It is OK to buy products of animals that have suffered if the price is low enough
22.7 Animals should be unconscious (stunned) before they are killed
22.8 Animals should be killed before being cooked
22.9 It is important to have legislation that ensures animal care is adequate
22.10 Animal protection organization are important in ensuring animals are adequately cared for
